# Identification of OP354-like human rotavirus strains with subtype P[8]b in Ghanaian children with diarrhoea

**DOI:** 10.1186/s12985-016-0523-5

**Published:** 2016-04-22

**Authors:** Susan Damanka, Francis E. Dennis, Chantal Agbemabiese, Belinda Lartey, Theophilus Adiku, Kofi Nyarko, Christabel C. Enweronu-Laryea, Kwamena W. Sagoe, Michael Ofori, Onike Rodrigues, George E. Armah

**Affiliations:** Department of Electron Microscopy and Histopathology, Noguchi Memorial Institute for Medical Research, College of Health Sciences, University of Ghana, Legon, Accra, Ghana; Department of Microbiology, University of Ghana Medical School, College of Health Sciences, Accra, Ghana; Department of Epidemiology and Disease Control, School of Public Health, College of Health Sciences, University of Ghana, Legon, Accra, Ghana; Department of Child Health, Korle Bu Teaching Hospital, College of Health Sciences, University of Ghana, Accra, Ghana; Department of Immunology, Noguchi Memorial Institute for Medical Research, College of Health Sciences, University of Ghana, Legon, Accra, Ghana

**Keywords:** Rotavirus, P[8]b subtype, Ghana, Nucleotide sequence, Phylogenetic analysis

## Abstract

**Background:**

Rotaviruses with the P[8] genotype have been associated with majority of infections. Recent improvements in molecular diagnostics have delineated the P[8] genotype into P[8]a and P[8]b subtypes. P[8]a is the previously known P[8] genotype which is common whilst P[8]b subtype also known as OP354-like strain is genetically distinct, rarely detected and reported from a few countries. In a previous study, the P-types could not be determined for 80 RVA-positive samples by conventional RT-PCR genotyping methods with the recommended pool of P-genotype specific primers used in the WHO Regional Rotavirus Reference Laboratory in Ghana. The present study employed sequence-dependent cDNA amplification method to genotype previously non-typeable P-types.

**Methods:**

Viral RNAs were extracted and rotavirus VP4 genes amplified by one step RT-PCR using gene specific primers. PCR amplicons were purified, sequenced and sequences aligned with cognate gene sequences available in GenBank using the ClustalW algorithm. Phylogenetic analysis was performed using the Neighbour-Joining method in MEGA v6.06 software. Phylogenetic tree was statistically supported by bootstrapping with 1000 replicates, and distances calculated using the Kimura-2 parameter model.

**Results:**

Of the 80 RVA-positive samples, 57 were successfully sequenced and characterized. Forty-eight of these were identified as P[8] strains of which 5 were characterized as the rare P[8]b subtype. Phylogenetic analysis of the VP8* fragment of the VP4 genes of these P[8]b strains revealed a close relationship with prototype OP354-like P[8]b strain and P[8]b strains of Russian and South African P[8]b origin.

**Conclusion:**

The study highlights the importance of regularly updating the primers employed for molecular typing of rotaviruses.

## Background

Group A rotaviruses are the most important aetiological agent of severe dehydrating diarrhoea in children less than 5 years of age accounting for an estimated 453,000 deaths annually [1]. Rotaviruses with the P[8] genotype have been associated with majority of these infections, accounting for approximately 74 % of global prevalence [2]. In order to control severe disease caused by rotavirus infection, vaccination is considered an essential strategy. The two currently available rotavirus vaccines (Rotarix™ and RotaTeq™) both include the rotavirus P[8] genotype, which has recently been classified into two subtypes- P[8]a and P[8]b (OP354-like) [3, 4]. Whilst P[8]a which corresponds to the previously known P[8] genotype is common, P[8]b is rarely detected, genetically distinct from P[8]a with limited reports globally [4–6]. The prototype OP354 strain bearing P[8]b specificity was detected in the Malawian strain [6]. A recently inferred evolutionary history of OP354-like P[8]b rotavirus strains globally showed that these strains emerged relatively recently, spreading from South and East Asia to Europe, Sub-Saharan Africa and North America [7]. In previous WHO sponsored rotavirus surveillance studies in selected African countries, [8] the genotypes of 80 RVA-positive samples with first round RT-PCR amplification products could not be determined with the recommended primers presently available within the WHO Regional Reference Laboratory in Ghana. This study sought to determine genotypes of previously non-typeable rotavirus VP4 genes using sequence-dependent cDNA amplification methods.

## Results

**Fig. 1:**
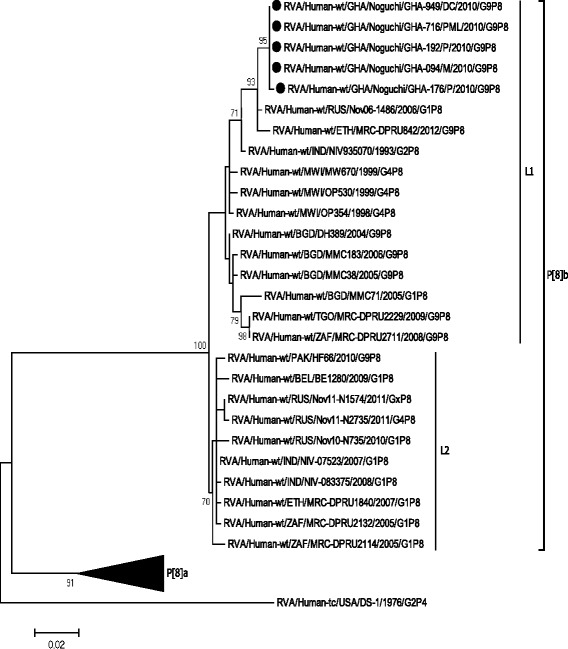
Phylogenetic tree of the VP8* fragment of the VP4 genes of Ghanaian P[8]b strains (637 nt), P[8]a and published P[8]b strains constructed by the neighbor-joining method with MEGA 6.06 software and rooted with the human rotavirus strain, DS-1. Variation scale (nucleotide substitution per site) is indicated below the phylogenetic tree. Percentage bootstrap support is indicated by values at each node, and values ≥70 % are shown. P[8]b strains analysed in the present study are indicated by closed circles. Reference sequences used in the analysis were obtained from the GenBank database. Phylogenetic distance was measured by Kimura two-parameter model. Phylogenetic trees were supported statistically by bootstrapping with 1000 replicates

RVA-positive samples (*n* = 80) whose VP4 genes could not be characterised using the pool of genotype specific primers recommended for use in the WHO Regional Reference Laboratory in Ghana were included in the study. Fifty-seven of the RVA-positive samples were successfully sequenced and characterised using the web-based genotyping tool RotaC version 2.2 [9]. Forty-eight (84.2 %) of these were characterised as P[8] strains of which 43 (89.6 %) were identified as P[8]a and 5 (10.4 %) as the rare OP354-like P[8]b subtype. The 5 P[8]b subtypes detected in the study were found in combination with G9 genotype. Sequence mismatches were detected in the primer binding region of the target gene of the 43 Ghanaian P[8]a rotavirus strains when aligned with the available WHO P[8]a primers (1 T-1, 1 T-1Wa and 1 T1-VN) which might have resulted in genotyping failure (data not shown). The Ghanaian P[8]b rotavirus strains (GHA-949/DC/2010, GHA-716/PML/2010, GHA-094/M/2010, GHA-176/M/2010 and GHA-192/M/2010) shared more than 99 % nucleotide sequence identity amongst themselves and with the Russian P[8]b strain (Rus/Nov06-1486). However, they shared a slightly lower nucleotide identity (96.3–98.9 %) with other published African P[8]b strains (Table 1). The Ghanaian strains formed a monophyletic cluster within lineage I of the P[8]b subtype, clustering along with the prototype strain OP354 and other published strains from Africa (Ethiopia, Malawi, Togo South Africa), Asia (India, Bangladesh, Pakistan) and Eastern-Europe (Russia) (Fig. 1).

**Table 1:**
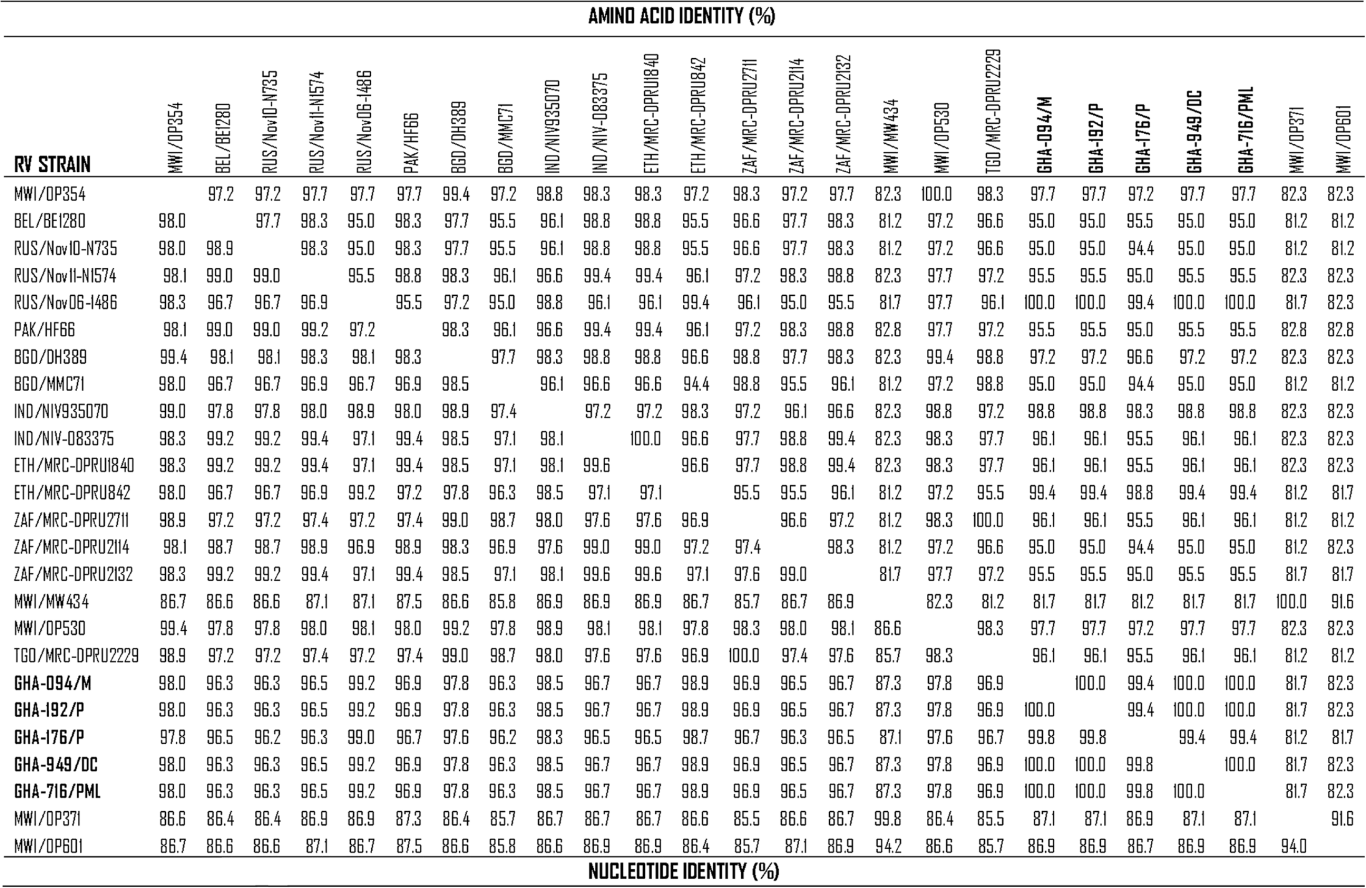
Comparison of the Deduced Amino acid (right, upper) and Nucleotide (left, below) identities of the VP8* fragment of VP4 gene of rotavirus P[8]b from Ghana with other P[8]b strains from the GenBank. Study isolates are marked in boldface type

## Discussion

Rotavirus continues to be a major cause of diarrhoeal disease among children less than 5 years old. Among the five major rotavirus strains (G1P[8], G2P[4], G3P[8], G4P[8] and G9P[8]) causing severe disease in children, G1P[8] has been found to be the predominant strain [2, 10]. Rotaviruses with the P[8] VP4 genotype are a major cause of acute infantile diarrhoea. Recent improvement in molecular characterisation has delineated the P[8] genotype into P[8]a and P[8]b subtypes. The P[8]a subtype is the previously known common P[8]genotype, whilst the P[8]b (OP354-like P[8]) subtype is rare and has been identified in only a few cases [5]. In this study, we report for the first time rotaviruses with the P[8]b subtype as a cause of diarrhoeal disease in hospitalized Ghanaian children. The prototype P[8]b rotavirus (strain OP354) was reported in Malawi, Blantyre during a two year study of viral gastroenteritis in children (1997–1999) [6] and subsequently detected mainly in Asian countries [11–13]. The Ghanaian OP354-like P[8] strains were phylogenetically closely related to strains mostly from Africa and South Asia suggesting common origin. This finding supports the recently inferred evolutionary history of OP354-like P[8]b rotavirus strains globally [7]. Compared with other VP4 P[8] lineages, strains bearing OP354-like P[8] genes emerged relatively recently, spreading from South and East Asia to Europe, Sub-Saharan Africa and North America in less than 20 years. Considering that all the Ghanaian OP354-like P[8] strains were isolated in the same rotavirus season, it is unclear whether they are continually circulating or their detection resulted from a one-time seeding event. Continuous surveillance of rotavirus strains using updated PCR primer sets capable of detecting OP354-like P[8] strains should clarify this, and in the process, determine whether they will be an important genotype in the human rotavirus population. Though, Nguyen et al., [11] speculated that P[8]b rotaviruses cause more severe disease than P[8]a subtypes, this study was unable to validate this position or otherwise. This was due to the disproportionately small number of P[8]b strains as well as insufficient clinical records. In the present study, 10.4 % (5/48) of previously non-typeable P[8] rotaviruses were successfully sequenced and characterized as the rare OP354-like P[8]b rotavirus strain. Both of the currently available rotavirus vaccines [Rotarix™ and RotaTeq™] include P[8]a VP4 gene but not P[8]b [3, 14]. It has been reported that there is a relatively large genetic distance between OP354-like P[8] strains and the P[8]a strain contained in both vaccines which is translated into multiple differences in antigenic epitopes [7]. Therefore, it remains unclear whether these vaccines will provide efficient protection against P[8]b rotavirus strains [4, 15]. Full genome characterization of the Ghanaian P[8]b subtype is presently being conducted to elucidate their origin and evolutionary dynamics.

## Conclusion

We have been able to characterize the VP4 genes of previously non-typeable Ghanaian rotavirus genotypes and established their phylogeny. The rare OP354-like P[8]b subtype of rotavirus strain has been detected and reported for the first time in Ghana. Expansion of P[8]b subtype data would lead to a better understanding of transmission dynamics of this rare subtype in Ghana in relation to P[8]a. The study highlights the importance of regularly updating PCR primers employed for molecular typing of rotaviruses. It also indicates the necessity for continuous surveillance especially in the post-vaccine era.

## Methods

The study was reviewed and approved by the Institutional Review Board, Noguchi Memorial Institute for Medical Research, Legon, Accra, Ghana.

Viral RNA was extracted from 10 % stool suspensions by the phenol-chloroform method [16] and purified with the RNaid® Kit (Bio 101, Carlsbad, USA). A one step RT-PCR targeting the VP4 genes of RVA was carried out using the PCR primer pairs, Con2/Con3 and VP4-F/VP4-R to generate 876 bp and 663 bp VP8*gene fragments respectively. The success of gene amplification was verified by electrophoresis on 2 % agarose gel stained with ethidium bromide. The VP4-F/VP4-R RT-PCR products (663 bp) were purified with the QIAquick PCR purification kit (Qiagen/Westburg) following the manufacturer’s instructions. Sequencing of the purified products was performed using the di-deoxynucleotide chain termination method with the ABI PRISM™ BigDye Terminator Cycle Sequencing Reaction Kit v.3.1 (Applied Biosystems, Foster City, CA, USA). After post purification, sequences were read in an automated sequencer (ABI PRISM™ 3130).

### Sequence analysis

Consensus sequences determined from forward and reverse sequences were used to query the nucleotide database in GenBank using the Basic Local Alignment Search Tool [BLAST] (http://blast.ncbi.nlm.nih.gov/Blast.cgi). Genotypes were confirmed using the automated genotyping tool, RotaC v2.0 [9]. Sequences were edited, trimmed to the same length to maximise homology and homology table was constructed using Bioedit v. 7. 0. 5 (Table 1). Nucleotide sequences of confirmed rotavirus genotypes were aligned with cognate gene sequences available in GenBank using the ClustalW algorithm [17], and phylogenetic analysis was performed using the Neighbour-Joining method in MEGA v6.06 software [18, 19]. Phylogenetic tree was statistically supported by bootstrapping with 1000 replicates, and phylogenetic distances calculated using the Kimura-2 parameter model. The Ghanaian P[8]b and reference P[8]b strains used in the construction of the phylogenetic tree are listed in Table 2.

**Table 2:** Rotavirus P[8]b study strains and published P[8]b reference strains with their GeneBank accession numbers used in the construction of the phylogenetic tree

Reference Strain	Accession Number
RVA/Human-wt/BEL/BE1280/2009/G1P8	JN849149.1
RVA/Human-wt/BGD/DH389/2004/G9P8b	GQ869838.1
RVA/Human-wt/BGD/MMC38/2005/G9P8b	EU979379.1
RVA/Human-wt/BGD/MMC183/2006/G9P8b	GQ869840.1
RVA/Human-wt/BGD/MMC71/2005/G1P8b	EU979382.1
RVA/Human-wt/ETH/MRC-DPRU842/2012/G9P8	KJ753482.1
RVA/Human-wt/ETH/MRC-DPRU1840/2007/G1P8	KJ753382.1
RVA/Human-wt/IND/NIV-07523/2007/G1P8	HQ881574.2
RVA/Human-wt/IND/NIV935070/1993/G2P8	DQ887043.2
RVA/Human-wt/IND/NIV-083375/2008/G1P8	HQ881575.2
RVA/Human-wt/MWI/MW670/1999/G4P8	KP902535.1
RVA/Human-wt/MWI/OP530/1999/G4P8	KP902533.1
RVA/Human-wt/MWI/OP354/1998/G4P8	KP902534.1
RVA/Human-wt/PAK/HF66/2010/G9P8	JX273730.1
RVA/Human-wt/RUS/Nov06-1486/2006/G1P8	FJ435210.2
RVA/Human-wt/RUS/Nov10-N735/2010/G1P8	HQ537508.2
RVA/Human-wt/RUS/Nov11-N2735/2011/G4P8	JX682949.2
RVA/Human-wt/RUS/Nov11-N1574/2011/P8	JX261756.1
RVA/Human-wt/TGO/MRC-DPRU2229/2009/G9P8	KJ752610.1
RVA/Human-wt/ZAF/MRC-DPRU2711/2008/G9P8	KJ752939.1
RVA/Human-wt/ZAF/MRC-DPRU2114/2005/G1P8	KJ752076.1
RVA/Human-wt/ZAF/MRC-DPRU2132/2005/G1P8	KJ753161.1
RVA/Human-wt/GHA-949/DC/2010	KM379145
RVA/Human-wt/GHA-716/PML/2010/G9/P8	KM379146
RVA/Human-wt/GHA-094/M/2010/G9/P8	KM379147
RVA/Human-wt/GHA-176/M/2010/G9/P8	KM379148
RVA/Human-wt/GHA-192/M/2010/G9/P8	KM379149

### Nucleotide sequence accession numbers

The nucleotide sequences of the P[8]b rotaviruses reported in the study have been deposited in the GenBank database under accession numbers KM379145-KM379149 (GHA-949/DC/2010; GHA-716/PML/2010; GHA-094/M/2010; GHA-176/M/2010 and GHA-192/M/2010 respectively).
